# Investigation of the Impacts of Antibiotic Exposure on the Diversity of the Gut Microbiota in Chicks

**DOI:** 10.3390/ani10050896

**Published:** 2020-05-21

**Authors:** Abdelmotaleb A. Elokil, Khaled F.M. Abouelezz, Hafiz I. Ahmad, Yuanhu Pan, Shijun Li

**Affiliations:** 1 Key Laboratory of Agricultural Animal Genetics, Breeding and Reproduction, Ministry of Education, College of Animal Science and Veterinary Medicine, Huazhong Agricultural University, Wuhan 430070, China; abdelmotaleb@mail.hzau.edu.cn (A.A.E.); panyuanhu@mail.hzau.edu.cn (Y.P.); 2 Department of Animal Production, Faculty of Agriculture, Benha University, Moshtohor 13736, Egypt; 3 Department of Poultry Production, Faculty of Agriculture, Assiut University, Assiut 71526, Egypt; abollez@aun.edu.eg; 4 Department of Livestock Production, University of Veterinary and Animal sciences, Ravi Campus, Pattoki 55300, Pakistan; ishfaq.ahmad@uvas.edu.pk

**Keywords:** enrofloxacin, diclazuril, gut microbiome, metagenomics, chicken

## Abstract

**Simple Summary:**

Broad-spectrum antibiotics have been a cornerstone in the treatment of bacterial diseases. However, growing evidence suggests that antibiotics have effects on host-associated gut microbiota communities. In this study, we report persistent significant changes in the abundance of gut microbiota and their functional metabolite pathways in chickens due to enrofloxacin and diclazuril exposure. These changes may affect the taxonomic, genomic, and functional capacity of the chicken gut microbiota, reducing bacterial diversity while expanding and collapsing membership of specific indigenous taxa. Understanding the biology of competitive exclusion of adaptive functions during antibiotic exposure in the gut may inform the design of new strategies to treat infections, while preserving the ecology of chicken-beneficial constituents.

**Abstract:**

The dynamic microbiota in chickens can be affected by exposure to antibiotics, which may alter the composition and substrate availability of functional pathways. Here, 120 Jing Hong chicks at 30 days of age were randomly divided into four treatments totaling seven experimental groups: control chicks not exposed to antibiotics; and chicks exposed to enrofloxacin, diclazuril, and their mixture at 1:1 for 14 days and then not exposed for a withdrawal period of 15 days. Fecal samples were collected from the 7 groups at 8 time-points (exposure to 4 antibiotics and 4 withdrawal periods) to perform in-depth 16S rRNA sequencing of the gut microbiota. Taxon-independent analysis showed that the groups had significantly distinct microbial compositions (*p* < 0.01). Based on the microbial composition, as compared with the control group, the abundances of the phyla *Firmicutes*, *Actinobacteria*, *Thermi*, and *Verrucomicrobia*, as well as the families *Lactobacillus*, *Lactococcus*, *S24-7*, and *Corynebacterium*, were decreased in the antibiotic-exposed chicks (*p* < 0.01). Phylogenetic Investigation of Communities by Reconstruction of Unobserved States (PICRUSt) analyses revealed significant differences in microbiota metabolite pathways due to the genera of the antibiotic-responsive microbes (*p* < 0.01), especially the pathways relating to cell growth and death, immune system diseases, carbohydrate metabolism, and nucleotide metabolism. Oral treatment with enrofloxacin, diclazuril, and their mixture modified the gut microbiota composition and the microbial metabolic profiles in chickens, with persistent effects (during the withdrawal period) that prevented the return to the original community and led to the formation of a new community.

## 1. Introduction

Anticoccidial products are often used to control diseases such as coccidiosis in flocks of chickens. However, only a few antibiotic drugs (such as enrofloxacin and diclazuril) have great efficacy against pre-existing infections, such as *Salmonella* spp., *Campylobacter*, and *Eimeria* spp. [1,2]. These antibiotic affect the gut microbiota and their metabolic pathways [3,4]. The desired scenario would be to kill the parasite but also to allow the development of the gut microbiome to enhance natural immunity [5]. Specifically, gut microbiota may alter the pathophysiology of parasite infections, and changes in microbiota can confer resistance to enteric protozoa or can promote protozoan infection, because normal or healthy microbiota decrease the host susceptibility to this parasite [6,7]. On the other hand, host metabolomics may enable global metabolite perturbations in response to the antibiotics mediated from gut secretion. These antibiotics have been found to alter the structural, compositional, and functional capacity of gut microbiota in antibiotic-exposed hosts [8]. Although antibiotic treatment in vitro and in vivo decreases not only the number of bacteria but also the diversity of the microbiota from days to weeks after the cessation of antibiotic administration, it is possible that some bacterial species could be permanently depleted from the community [9,10].

Enrofloxacin is a fluoroquinolone antibiotic that is currently approved by the US Food and Drug Administration (FDA) for use in water to treat flocks of poultry in order to promote the evolution of fluoroquinolone-resistant strains against Campylobacter pathogens [11]. In addition, diclazuril is a polyether antibiotic that is effective for treating infections caused by *Isospora* spp., *Toxoplasma gondii*, and *Eimeria* spp. [12]. One-day-old broiler chicken that were exposed to antibiotics for 24 hours revealed perturbations in the gut microbiota, which negatively affects intestinal immune development [13]. Furthermore, antibiotic treatment selects for resistant bacteria, increases opportunities for horizontal gene transfer, and enables intrusion of pathogenic organisms through depletion of occupied natural niches, with profound implications for the emergence of resistance [2,8].

Host and environmental factors influence the gut composition; comparative environmental factors (diet, medicines, and antibiotics) are more dominant in shaping the host microbiota than the host genotype [8,14]. Understanding the impacts of antibiotics on the host–microbe relationship, including the biology of competitive exclusion or the protection of microbiome taxa, as well as the gene flow of symbiotic functions in the gut ecology, may reveal safety strategies for the treatment of infections while preserving beneficial intestinal ecology [15,16]. Therefore, the objective of this study was to investigate the impact of antibiotic exposure (enrofloxacin and diclazuril) for two weeks followed by a two week withdrawal period on the composition and function of the normal microbial colonization of chickens.

## 2. Materials and Methods

### 2.1. Ethics and Approval Statement

The protocols for all animal experiments were approved by the Scientific Ethic Committee of Huazhong Agricultural University, approval number HZAUCH-2019-005. Chicks were handled in accordance with the guidelines described by the Animal Care Committee of Hubei Province, P.R. China.

### 2.2. Experimental Design and Fecal Sample Collection

A total of 120 Jing Hong chicks with similar genotypes, age (30 days old), and weights (280 ± 30 g) were used in this study. The chicks were not exposed to antibiotics or anticoccidial drugs before 30 days of age. To conduct the experiment, all chicks were randomly divided into 4 groups (30 chicks/group), including one control group and three experimental groups. All the chicks were obtained from the poultry farm of Huazhong Agricultural University, Hubei, China; the chicks were kept under the same conditions, housed in cages, and supplied with the same feed (standard caloric and nitric diet) and water during the entire experiment. 

**Figure 1:**
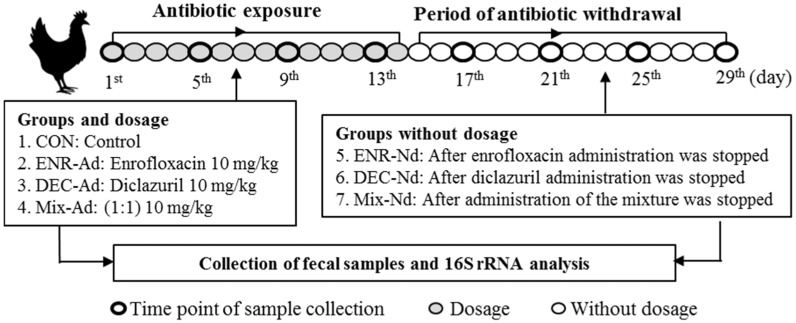
Animal experimental design; six experimental groups were obtained as follows: addition of enrofloxacin (ENR-Ad), no enrofloxacin (ENR-Nd), addition of diclazuril (DEC-Ad), no diclazuril (DEC-Nd), addition of drug mixture (MIX-Ad), and no drug mixture (MIX-Nd), while the seventh group was considered the control group.

The enrofloxacin and diclazuril were obtained from Qilu Animal Health Products Co., Ltd., Shandong, China (http://en.qiludb.com/product/48.html), and Jiangsu HFQ Bio-Technology Co., Ltd., Beijing, China (https://www.heaftron.com/veterinary-oral-solution/diclazuril-solution.html), respectively. Enrofloxacin at 10 mg/kg body weight (BW), diclazuril at 0.3 mg/kg BW, and a mix of enrofloxacin and diclazuril (1:1) were administered daily for 14 days. The drugs were added to the water boxes in the cages of the first, second, and third experimental groups. Then, we stopped the addition of drugs for another two weeks to obtain three subgroups of withdrawal periods for the drugs. The control group was not given any additives. The additional drugs were stopped after 14 days, at which point all chicks consumed pure water for another 15 days. At the end of the experimental period, all surviving chicks were sacrificed by decapitation. A total of 6 experimental groups were obtained as follows: addition of enrofloxacin (ENR-Ad), no enrofloxacin (ENR-Nd), addition of diclazuril (DEC-Ad), no diclazuril (DEC-Nd), addition of drug mixture (MIX-Ad), and no drug mixture (MIX-Nd), while the seventh group was considered the control group. Each chick from the seven groups was considered as an experimental unit. The fresh fecal samples were collected from the six experimental groups every 3 days at eight time points (4 times with the addition of drugs and 4 times without the addition of drugs), preserved in liquid nitrogen, and used for DNA extraction and PCR amplification. To investigate the impacts of antibiotic exposure on the gut microbiota diversity of chicks, a total of 4 samples were collected into clean tubes under cooling conditions from the ENR-Ad, ENR-Nd, DEC-Ad, DEC-Nd, MIX-Ad, and MIX-Nd groups, as well as 12 samples from the control group, and stored at −20 °C until DNA extraction. The animal experimental design is presented in Figure 1, in which the flowchart demonstrates the selection process for the data included in the analysis (Figure S1).

### 2.3. Microbial DNA Extraction, PCR Amplification, and 16S rRNA Analysis

The total genomic DNA of fecal digesta was extracted using a QIAamp DNA Stool Mini Kit (Qiagen GmbH, Hilden, Germany) according to the manufacturer’s instructions. To check the DNA quality before sequencing, the concentration, integrity, and purity of the extracted genomic DNA were measured using a Nanodrop device and 0.8% agarose gel electrophoresis. The DNA was quantified by UV spectrophotometer. Then, the extracted genomic DNA was used as a template. The variable region of 16S rRNA (V4 region) was amplified using the universal primers (341F,ACGCGGGTATCTAATCCTGTTTGCTCCCCACGCTTTCGCGCCTCAGTGTCAGTAC;802R,ABABADBBDFFFGGGFGGGFGGHGBGHGGHGGGGGGHGGGGGGGHHGGFBGEGGEG) [17]. The PCR conditions were as follows: initial denaturation, annealing, and extension were carried out and repeated at 94 °C for 4 min, 94 °C for 30 s, 50 °C for 45 s, and 72 °C for 30 s for 25 cycles. After confirming the sufficient quality of PCR products, library construction was conducted. Finally, PCR products were purified using a Quick Gel Extraction Kit (QIAGEN, cat 28706) according to the manufacturer’s instructions.

### 2.4. Sequence Quality and OTU Calculation

Amplified libraries of barcoded V4 were sequenced using the Illumina MiSeq sequencing platform, which included 160 bp paired-end reads that were generated with a 7-cycle index read. The sequence quality was determined after removing sequences with lengths less than 160 bp using Quantitative Insights Into Microbial Ecology (QIIME v1.8.0, http://qiime.org; Northern Arizona University, Flagstaff, AZ, USA) software, which required that the overlap of read 1 and read 2 be ≥ 10 bp and without any mismatches, according to [18]. The resulting sequences were clustered into operational taxonomic units (OTUs) using Uparse (Uparse v7.0.1001) at 97% sequence identity. Additionally, Specaccum analysis was applied to check whether all OTU abundance matrices were sufficient to estimate community richness (Figure S2). Finally, the phylogeny of OTUs as microbial diversity units, which usually refers to the sequence of one or more samples based on a sequence similarity threshold set by an individual, was calculated according to Blaxter et al. [19]. All sequence processing was performed by Shanghai Personal Biotechnology Co. Ltd., China, with the opening number MbPL201901330. Based on the V4 regions of the 16S rRNA sequences that passed the quality criteria, the average sequenced amplicon length was 160 bp. Data were generated at the species level using cutoffs for the parameter classification of 8 for the maximum e-value, 98% for minimum percentage identity, and 120 bp for minimum alignment length.

### 2.5. Annotation of Microbial Composition

Alpha diversity analyses, including the Shannon, Simpson, Chao1, and Abundance-based Coverage Estimators) ACE( indices, as well as the community uniformity, were applied to find the diversity of the microbiome communities among samples [20,21,22]. Beta diversity analysis for the Principal Component Analysis (PCA), Principal Coordinates Analysis (PCoA), Non-metric Multidimensional Scaling (NMDS), and Unweighted Pair Group Method with Arithmetic mean (UPGMA) clusters was used to obtain the comparative analysis of intergroup and group differences in terms of Unique Fraction (UniFrac) distance [23]. Heat map analysis was presented according to the top 50 most abundant distributions and the degree of similarity between the samples. The order analyses for Partial least squares discriminant analysis (PlS-DA), Adonis/PERMANOVA, and Analysis of similarities (ANOSIM) were performed to determine the variation in the community structure between groups by screening the key species. Metastat comparison of statistic tests among groups (http://metastats.cbcb.umd.edu/) was performed using Mothur software, and the quantity differences at the genus and phylum levels were estimated by pairwise comparison [24]. Linear discriminant analysis Effect Size (LEfSe) analysis based on linear discriminant analysis (LDA) was performed by submitting a relative abundance matrix at the genus level through the Galaxy online analysis platform (http://huttenhower.sph.harvard.edu/galaxy/) to obtain the candidate differences in the community composition [25]. UPGMA clustering analysis was performed on unweighted and weighted UniFrac distance matrices using QIIME software and visualized using R software.

### 2.6. Annotation of Microbial Function

To predict the bacterial metabolism function based on total genome sequences by the 16S rRNA gene, a functional predictive analysis of phylogenetic investigation of communities by reconstruction of unobserved states (PICRUSt) was performed to predict the metabolic function of bacteria and archaea [26]. PICRUSt can predict the associated functions of 16S rRNA gene sequences with three functional profile databases: Kyoto Encyclopedia of Genes and Genomes (KEGG), Clusters of Orthologous Groups (COGs), and RNA Family (Rfam). In particular, the KEGG pathway database (http://www.genome.jp/kegg/pathway.html) is classified into six categories, including metabolism, genetic information processing, environmental information processing, cellular processes, organismal systems, and human diseases, each of which is further divided into multiple levels. 

### 2.7. Accession Number

All raw data of microbial genomic sequencing were deposited at the National Center for Biotechnology Information (NCBI) and can be accessed in the BioProject (https://www.ncbi.nlm.nih.gov/bioproject/PRJNA601006) under the accession number PRJNA601006.

### 2.8. Statistical Analyses

Diversity index data were statistically analyzed using one-way analysis of variance (ANOVA) and significant differences among group means were determined using the least significant difference (LSD) test. All values for the diversity index and bacterial metabolism function are expressed as the means ± standard error of the mean (SEM). Nonmetric multidimensional scaling (NMDS) plots of sequence read abundance were generated with Vegan in R. All statistical analyses were performed using the General Linear Model (GLM) procedure of SAS (SAS Institute Inc., 2002, Cary, NC, USA, version 9). Individual chicks were considered as experimental units and one fixed effect (the duration of fertility) was included in the statistical model. All differences were considered significantly different at *p* < 0.05 and were indicated as trends when *p* < 0.10. Pairwise comparisons were performed using Duncan’s multiple range test. 

## 3. Results

### 3.1. Effective Sequence Quality Assessment

High-throughput sequencing generated from 36 individual chickens yielded a total of 1,659,563 reads (average of 46,098, ranging from 33,037 to 68,274 reads for each sample), as presented in Figure S3. The average read length was 160 bp, and the distributions of sequence lengths shown in OTUs were generated and characterized for different taxonomic levels, including the domain, phylum, class, order, family, and genus levels, based on the Greengene database using QIIME. Taxonomies present in samples were considered common and their abundance counts were used for further analysis. The statistical numbers of OTUs at each classification level among the control and antibiotic-exposed chicks are presented in Table S1. A total of 11 phyla, 20 classes, 31 orders, 66 families, 100 genera, and 42 species were identified in these samples (Table S2). The candidate phyla of *Firmicutes*, *Bacteroidetes*, *Actinobacteria*, *Proteobacteria*, *Cyanobacteria*, *Deferribacteres*, *Fusobacteria*,*Tenericutes*, *Verrucomicrobia*, and *Thermi*, as well as the families of *Lactobacillus*, *Lactococcus*, *Ruminococcus*, *Corynebacterium*, *Sphingobium*, *Fusobacterium*, *Muciniphila*, and *S24-7*, were found to be significantly different in terms of the relative abundance of microbial communities between control and test groups (Table 1 and Table S2). 

### 3.2. Impact of Antibiotics on the Microbial Diversity Analysis of Exposed Chicks

The total observed OTU counts and alpha diversity indicators of Simpson, Chao1, ACE, and Shannon indices among the groups are summarized in Table 2. The total observed OTUs were significantly (*p* < 0.01) different among the groups. Alpha diversity was compared among the seven groups (ENR-Ad, ENR-Nd, DEC-Ad, DEC-Nd, MIX-Ad, MIX-Nd, and CON), as presented in Table 2. All alpha diversity indicators were calculated based on the OTUs using the phylogenetic diversity indices ACE, Chao1, Shannon, and Simpson. The values of diversity indicators for both Shannon and Simpson indices were highest in the DEC-Ad group and lowest in the MIX-Ad group of antibiotic-exposed chickens. Additionally, the highest and lowest values of both Chao1 and ACE, indicators of species richness, were estimated in the ENR-Ad and CON groups, respectively (Table 2). 

**Figure 2:**
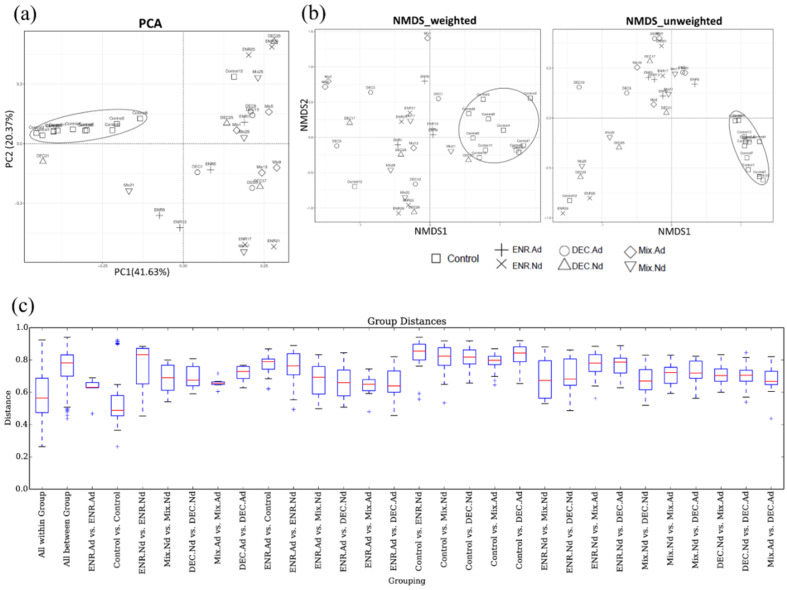
(**a**) Principal component analysis (PCA), (**b**) nonmetric multidimensional scaling (NMDS), and (**c**) boxplot for comparative analysis of intergroup–group differences in UniFrac distance pathways among groups of control and antibiotic-exposed chicks. ENR-Ad: addition of enrofloxacin; ENR-Nd: no enrofloxacin; DEC-Ad: addition of diclazuril; DEC-Nd: no diclazuril; MIX-Ad: addition of drug mixture; MIX-Nd: no drug mixture; CON: control group.

The beta diversity indicators (PCA, NMDS, and boxplot) were obtained to measure the intragroup and intergroup distances. A principal component analysis (PCA) based on the unweighted UniFrac distance is presented in Figure 2. To determine any separation into sample clusters, a PCA plot was constructed; the PCA plot revealed that compared to the samples corresponding to the control group, the gut microbiota of the antibiotic-exposed chickens were modulated (Figure 2a). Likewise, the beta diversity results of weighted and unweighted NMDS indicated the corresponding cluster distribution of the CON group and separated the distribution in another antibiotic-exposed group (Figure 2b). Statistically significant P values were obtained to measure the intragroup and intergroup distances; based on the boxplot, we found that the differences between groups were significantly (*p* < 0.01) higher than the differences within the groups of the observed species (Figure 2c). Both Adonis and ANOSIM analysis detected highly significant changes (*p* < 0.01) in the beta diversity among groups. The R^2^ values calculated by Adonis of weighted and unweighted UniFrac distances were 0.348 (*p* < 0.01) and 0.364 (*p* < 0.01), respectively. In addition, R^2^ values calculated by ANOSIM were 0.394 (*p* < 0.01) and 0.562 (*p* < 0.01) in weighted and unweighted UniFrac distances, respectively, which is consistent with the evident temporal structure of the data depicted in the PCA and NMDS plots.

### 3.3. Antibiotic-Exposed Chicks Alter their Gut Microbiota Community Structure

**Figure 3:**
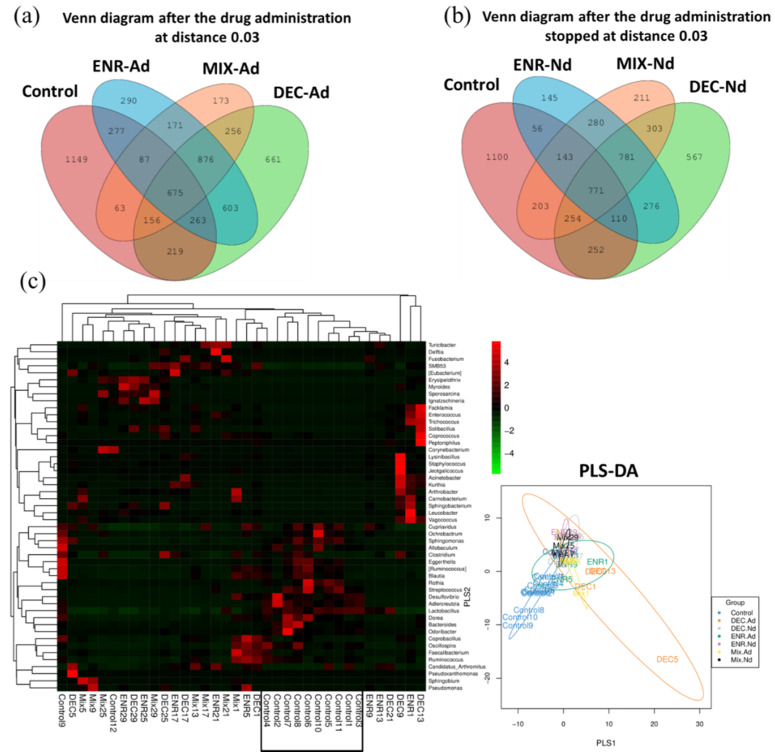
(**a**,**b**) Venn diagram of shared OTUs of the different groups during the period of drug administration (2 weeks) and during the period of no drug administration (2 weeks), respectively. The numbers below the groups indicate the number of OTUs within each sector. (**c**) Heatmap showing the genera with significant differences in relative abundances among the seven groups. Partial least squares discriminant analysis (PlS-DA) among groups of control and antibiotic-exposed chicks. ENR-Ad: addition of enrofloxacin; ENR-Nd: no enrofloxacin; DEC-Ad: addition of diclazuril; DEC-Nd: no diclazuril; MIX-Ad: addition of drug mixture; MIX-Nd: no drug mixture; CON: control group.

The proportions of common and unique OTUs among the CON, ENR-Ad, DEC-Ad, and MIX-Ad groups are presented in a Venn diagram, as shown in Figure 3a. Likewise, the OTU counts among the CON, ENR-Nd, DEC-Nd, and MIX-Nd groups are presented in Figure 3b. Additionally, the heat map of the top 50 most abundant compositions in the microbiome community combined with their cluster analysis showed similar microbiome compositions among samples of the control group in comparison with the six groups of antibiotic-exposed chickens (Figure 3c). There were wide variations in the bacterial taxa among groups (Table 1). The relative abundance of the phyla *Firmicutes*, *Proteobacteria*, and *Actinobacteria* collectively made up more than 90% of the total gut microbiota in each group, with *Bacteroidetes*, *Fusobacteria*, and *Cyanobacteria* present as minor constituents. *Firmicutes*, *Proteobacteria*, and *Actinobacteria* were the most abundant phyla in the MIX-Nd, MIX-Ad, and CON groups, respectively (Table 1). At the genus level, *Lactobacillus*, *Erysipelothrix*, *Acinetobacter*, and *Enterococcus* were common, and the highest abundance appeared in the CON, ENR-Ad, MIX-Ad, and DEC-Ad groups, respectively (Table 1). The relative abundances of *Lactobacillus*, *Streptococcus*, and *Ruminococcus* were higher in the control group than in the six antibiotic-exposed groups, while *Enterococcus* and *Acinetobacter* were higher in the six antibiotic-exposed groups than in the control group (Table 1).

### 3.4. Microbiota that Associate with Antibiotic-Exposed Chicks

**Figure 4:**
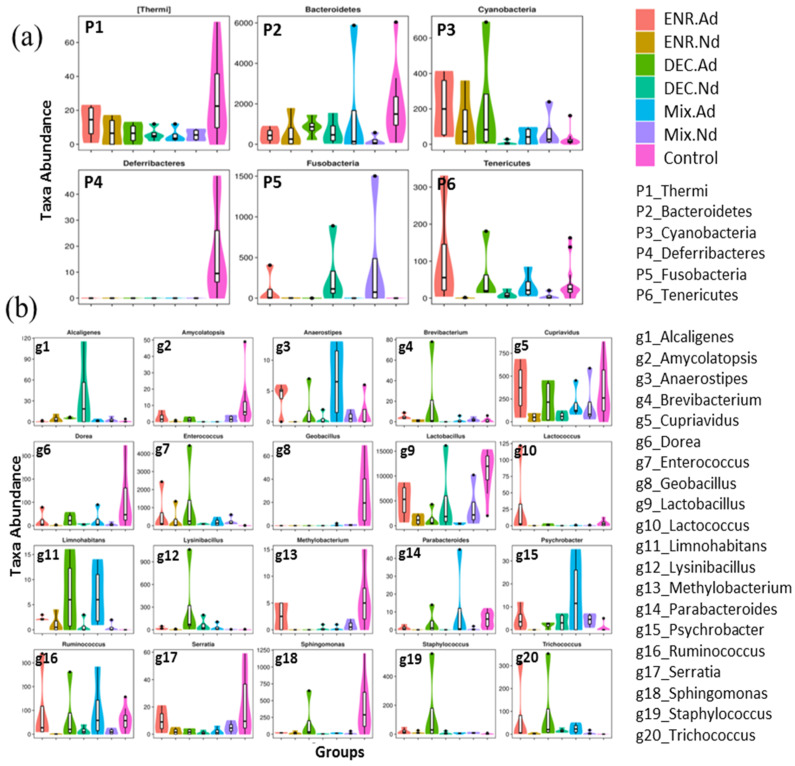
Metastat comparison statistics tests; (**a**) for phylum and (**b**) for genus among the control group and antibiotic-exposed chick groups.

To evaluate the similarity between samples, the unweighted pair-group method with arithmetic (UPGMA) analysis was performed, which indicated that samples of the CON group were mostly similar and distributed into one cluster of hierarchical trees (Figure S4). Moreover, to identify specific bacterial taxa that are associated with the responses of antibiotic-exposed chickens, we compared the fecal microbiota among groups by using LEfSe analysis based on linear discriminant analysis (LDA). A LEfSe cladogram representative of the structure of the host microbiota axis showed a significant shift in the microbiota among groups, including a total of 37 bacterial taxa that were significantly different among the seven groups, especially the *Lactobacillus*, *Streptococcus*, *Enterococcus*, *Acinetobacter*, and *Ruminococcus* families (Figure S5). The LDA score plot shows that group-enriched taxa were significant at *p* < 0.05 (Figure 3b). The abundance comparison among groups at the phylum and genus levels was performed by Metastats analysis (Figure 4). The six phyla of *Thermi*, *Bacteroidetes*, *Cyanobacteria*, *Deferribacteres*, *Fusobacteria*, and *Tenericutes* were the most abundant, with significant differences (*p* < 0.05) among groups. At the genus level, a total of 20 genera appeared, with significant differences (*p* < 0.05) among groups, including *Amycolatopsis*, *Dorea*, *Geobacillus*, *Methylobacterium*, *Serratia*, and *Sphingomonas*, which had the greatest differences compared with the CON group (Figure 4). In addition, the statistical results of the Metastats comparison tests between each group are presented in Table S3. The highest variation (5 phyla and 40 genera) was recorded between CON and ENR.Nd, whereas only one genus appeared in the comparison between DEC.Nd and Mix.Nd groups (Table S3).

### 3.5. Comparison of the KEGG Pathways of the Gut Microbiota among Groups of Antibiotic-Exposed Chicks

The microbial function prediction analysis was conducted through PICRUSt to determine the differences in the functions of microbiota among groups. Numerous functions are involved in metabolic pathways. At KEGG level 2, several metabolism pathways were elevated in the six groups of enrofloxacin- and diclazuril-exposed chickens, including amino acid metabolism, biosynthesis secondary metabolites, lipid metabolism, and xenobiotic biodegradation compared with the control group (Table 3). The pathways belonging to cellular processes showed highly significant (*p* < 0.01) differences among groups, whereas the differences in pathways of environmental information processing were not significant, except for membrane transport (Table 3). The top 50 abundance results for KEGG orthologous genes were clustered into a heat map, combined with analysis among groups of control and antibiotic-exposed chicks, as presented in Table 3. This included samples of control groups that appeared in relative clusters based on their similarity of microbial composition.

## 4. Discussion

Although antibiotics have been investigated for their activity against indigenous pathogenic bacteria, the collateral damage of host-associated microbiota communities still requires attention. These drugs have been correlated with alterations in both the structure and function of gut microbiota, with temporary effects (during the withdrawal period) that return to the originating community and sometimes persist, forming a new community [27]. Here, we report persistent significant changes in the abundance of gut microbiota and their functional metabolite pathways in chickens due to enrofloxacin and diclazuril exposure. Hence, the current study was chosen to assess the changes in the gut microbial metabolome to identify functional pathways, which may explain the previous observations.

Our results and those of others clearly indicate that there are substantial changes in gut community taxonomic composition in response to the administration of two antibiotics; therefore, we anticipated robust and wide alterations in the gut microbiome. The significant differences in the relative abundance of microbial communities between control and test groups, including *Lactobacillus*, *Streptococcus*, and *Ruminococcus*, were found to be higher in the control group, and *Enterococcus* and *Acinetobacter* were predominant in test groups. Enrofloxacin and diclazuril are broad-spectrum antibiotics that lead to reduced bacterial diversity, especially for *Lactobacillus*, *Streptococcus*, and *Ruminococcus*, while expanding and collapsing the candidate symbiotics of indigenous microbiome taxa [8,28]. Additionally, the host–microbiome interaction during antibiotic-mediated resistance was selected, increasing the opportunities for horizontal gene transfer and enabling the intrusion of pathogenic bacteria due to depletion of occupied natural niches, which suggests the emergence of resistance [16,29]. Precisely, these pervasive alterations can be viewed as a coupling of mutualistic host–microbe relationships. Therefore, it is valuable to reconsider antimicrobial therapies in the context of an environmental perspective.

In the present study, alpha and beta diversity indicators revealed higher significant differences among the seven groups compared to the intra groups (Table 2, Figure 2c). Indeed, the abundance of fecal *Firmicutes* increased in control groups at the expense of *Bacteroidetes* (*Firmicutes*/*Bacteroidetes* ratio) as compared with the antibiotic-exposed groups. *Firmicutes* and *Bacteroidetes* dominate the chicken fecal microbiota [30]. These phyla are linked to nutrient consumption, and consequently to energy harvest from the diet. In contrast, decreased exposure to antibiotics increased the abundance of fecal *Bacteroidetes* at the expense of *Firmicutes*. Previously, the *Firmicutes* members in hosts exposed to antibiotics, especially enrofloxacin and diclazuril, decreased with respect to microbial recolonization after the withdrawal period [13,31].

The results also show that the composition of fecal microbiota in the control group was apparently in a different cluster than the other clusters of antibiotic-exposed chickens (Table 1, Figure 3c). The difference may be attributed to the access of antibiotic-exposed chicks; these chicks had access to an abundance of a broad-spectrum antibiotics and were able to reshape the microbiota community, directly affecting the composition of the gut microbiota, increasing the *Bacteroidetes* content, and lowering the *Firmicutes*/*Bacteroidetes* ratio compared with the control group. A study on a group of healthy human volunteers showed that a short course of oral ciprofloxacin failed to recover several bacterial taxa and decreased the diversity and richness of the microbiota community following antibiotic treatment [27]. Likewise, mouse models have revealed that treatment with enrofloxacin causes long-lasting changes in the composition of the microbiota that persist after the antibiotic withdrawal period [32].

The major findings of our study indicate that both of the administered antibiotics (enrofloxacin and diclazuril) had relative effects on the composition of the microbiota of chicks compared with the control group, as presented in the LEfSe cladogram (Figure S5). In addition, among the key phylotypes in the gut microbiome that were modulated by exposure to antibiotics, the Metastat analysis uncovered several putative phyla and genera that were significant based on LDA analysis, including the *Thermi*, *Bacteroidetes*, *Cyanobacteria*, *Deferribacteres*, *Fusobacteria*, and *Tenericutes* phyla. At the genera level, twenty genera were identified as being significantly different between the seven groups belonging to these phyla. Moreover, *Firmicutes* dominated the gut microbiota, with the Lactobacillus genus being the most prevalent [33]. The exposure to antibiotics did affect the abundance of *Firmicutes* in the present study, as has been reported previously [34]. Wide changes in the intestinal digesta microbial communities may be due to differential substrate abundance in the intestine among groups.

For the microbial function-wide effects of antibiotics, we show that oral administration of enrofloxacin and diclazuril disturbs the natural composition of gut microbiota and induces or represses different pathway responses. These observations indicated long-lasting modifications of the gut microbiota composition that persisted after the antibiotic withdrawal period and were uncoupled in the host–microbiome relationship. PICRUSt analysis aims to predict the unobserved character states of phylogenetic microbiota data regarding the prediction of functional pathways of the community. With respect to PICRUSt functional profiles, the oral dosage of enrofloxacin and diclazuril treatment showed significant effects on various biosynthetic pathways in the chicken gut microbiota. Therefore, the metabolism pathways of amino acid metabolism, biosynthesis secondary metabolites, lipid metabolism, and xenobiotic biodegradation were elevated in the six groups of enrofloxacin- and diclazuril-exposed chickens.

Carbohydrate metabolism and glycan biosynthesis pathways were reduced in these groups due to the disruption in the *Firmicutes*/*Bacteroidetes* ratio. Interestingly, all cellular process pathways (cell growth and death, cell motility, and transport and catabolism) in the six groups of enrofloxacin- and diclazuril-exposed chickens were increased compared with the control group, indicating that the efficiency was regulated by microbiota-resistant drugs. Environmental information processing and membrane transport signaling were increased in the control groups, while the decrease in the signal transduction pathway might be due to activated extracellular signaling molecules under antibiotic-mediated treatment. In addition, the functions associated with genetic information processing, such as DNA functions (sorting and degradation of proteasome, replication and repair, transcription machinery, and RNA transport), were consistently detected much less frequently in the control groups. This finding was expected, since the bactericidal activity and phage mobility are mainly affected by antibiotic-mediated targeting of DNA gyrase and DNA topoisomerase, which facilitate DNA replication, recombination, and transcription [29].

The host–microbiome–antibiotics axis leads to horizontal gene transfer in the gut environment through an available cache of genetic information, which takes advantage of the functional pathways of the altered gut community through genetic exchange [35]. Additionally, phage mobility effects from the response of bacterial DNA damage under antibiotic-mediated conditions enhances the connectivity of phage bacterial networks, potentiating some microbial resistance due to access to the phage metagenome [36]. Indeed, the development of antibiotic resistance in the microbiota is a result of the mutability of their genomes, which increases drug resistance in ecosystems. Therefore, the human microbiome has recently been found to serve as an impressive reservoir of antibiotic resistance genes [37,38]. The administration of a broad-spectrum antibiotic enhances host resistance genes, which may lead to an uncoupling of mutualistic relationships that have evolved over long periods of time among the host and its gut microbiota [28,39]. Thus, we noted highly significant differences between the control group and the six groups of antibiotic-exposed chicks. Moreover, the microbiome is a reservoir of antibiotic resistance for long periods of time; although subjects did not receive additional doses throughout the experimental study, their gut microbiota continued to harbor comparable levels of the resistance genes after four years [9].

## 5. Conclusions

In summary, this study shows that oral treatment with enrofloxacin, diclazuril, or a mixture of the two modifies the gut microbiota composition and metabolic profiles in chickens, with persistent effects (during the withdrawal period) that prevented the return to the original community and led to the formation of a new community. By performing this extensive integrated analysis of the gut microbiota, we have identified the abundance of gut microbial associations in antibiotic-exposed chicks and are now able to refine our understanding of the findings relating to microbiome–antibiotic interactions, which could be useful for studies in other host–antibiotic models.

## Figures and Tables

**Table 1:** Relative abundances of fecal microbiota and classification taxa among control and antibiotic-exposed chick groups estimated using the metagenomics analysis.

Phulum and Genuse ^1^	Enrofloxacin (ENR)		Declazuril (DEC)		Mix 1:1 (MIX)		Control	SEM	*p*
	ENR.Ad	ENR.Nd	DEC.Ad	DEC.Nd	Mix.Ad	Mix.Nd	CON		
*Firmicutes (P)*	76.840 ^bc^	73.530 ^c^	64.880 ^d^	73.530 ^c^	50.670 ^f^	78.740 ^b^	83.980 ^a^	1.530	0.001
*Lactobacillus*	1.960 ^d^	0.580 ^f^	0.700 ^e^	2.170 ^c^	0.200 ^g^	2.330 ^b^	5.520 ^a^	0.020	0.001
*Lactococcus*	0.030 ^c^	0.012 ^b^	0.002 ^c^	0.003 ^c^	0.020 ^d^	0.02 ^d^	0.087 ^a^	0.000	0.001
*Enterococcus*	0.300 ^a^	0.006 ^f^	0.300 ^b^	0.031 ^e^	0.087 ^c^	0.061 ^d^	0.009 ^g^	0.000	0.001
*Ruminococcus*	0.040 ^b^	0.003 ^c^	0.037 ^a^	0.007 ^c^	0.041 ^a^	0.005 ^d^	0.029 ^b c^	0.000	0.001
*Facklamia*	0.060 ^c^	0.020 ^e^	0.160 ^a^	0.05 ^d^	0.070 ^b^	0.010^f^	0.001 ^g^	0.001	0.001
*Arthromitus*	0.391 ^d^	0.0567 ^b^	0.774 ^a^	0.361 ^e^	0.480 ^c^	0.174^f^	0.178 ^f^	0.004	0.001
*Clostridium*	0.005 ^c^	0.005 ^c^	0.008 ^a^	0.007 ^b^	0.004 ^c d^	0.003 ^d^	0.003 ^d^	0.000	0.001
*Erysipelothrix*	0.179 ^e^	2.444 ^a^	0.272 ^d^	1.850 ^b^	0.258 ^d^	1.082 ^c^	0.183 ^e^	0.183	0.011
*Bacteroidetes (P)*	10.870 ^a^	8.120 ^b^	4.880 ^b^	7.720 ^b^	7.080 ^b^	7.610 ^b^	11.770 ^b^	2.260	0.090
*S24-7*	0.014 ^c^	0.008 ^e^	0.009 ^d^	0.019 ^b^	0.009 ^d^	0.009 ^d^	0.078 ^a^	0.001	0.001
*F/B ratio*	4.590 ^b^	12.270 ^a b^	14.840 ^a^	13.380 ^a^	7.090 ^a b^	6.600 ^a b^	7.760 ^a b^	2.450	0.050
*Actinobacteria (P)*	3.970 ^c^	2.950 ^d^	3.880 ^c^	3.690 ^c^	1.860 ^e^	4.440 ^b^	9.910 ^a^	0.140	0.001
*Corynebacterium*	0.023 ^d^	0.007 ^e^	0.031 ^c^	0.008 ^e^	0.002^f^	0.310 ^a^	0.087 ^b^	0.001	0.001
*Arthrobacter*	0.0290 ^c^	0.0140 ^d^	0.0760 ^b^	0.0120 ^e^	0.107 ^a^	0.008^f^	0.001 ^g^	0.003	0.001
*Bifidobacterium*	0.008 ^b^	0.006 ^c^	0.006 ^c^	0.009 ^d^	0.006 ^b^	0.008 ^b^	0.001 ^e^	0.000	0.001
*Proteobacteria (P)*	4.130 ^b^	4.240 ^b^	5.430 ^a^	5.810 ^a^	4.340 ^b^	1.510 ^c^	0.330 ^d^	1.350	0.040
*Sphingobium*	0.112 ^b^	0.023 ^c^	0.097 ^b^	0.077 ^d^	3.871 ^a^	0.012 ^c^	0.003 ^e^	0.001	0.001
*Pseudomonas*	0.259 ^b^	0.025 ^e^	0.13 ^b^	0.074 ^c^	0.339 ^a^	0.040 ^d^	0.003 ^g^	0.000	0.050
*Acinetobacter*	0.352 ^b^	0.028 ^c^	1.030 ^a^	0.348 ^b^	0.022 ^c d^	0.0130 ^d^	0.016 ^d^	0.003	0.001
*Cyanobacteria (P)*	0.960 ^a^	0.560 ^b^	0.970 ^a^	0.030^f^	0.210 ^d^	0.340 ^c^	0.103 ^e^	0.006	0.020
*MLE1-12*	0.008 ^b^	0.008 ^b^	0.009 ^a^	0.001 ^c^	0.008 ^b^	0.008 ^b^	0.008 ^b^	0.001	0.050
*Streptophyta*	0.103 ^a^	0.062 ^c^	0.101 ^b^	0.004 ^g^	0.009 ^e^	0.036 ^d^	0.007 ^f^	0.001	0.001
*Chloroflexi (P)*	0.004 ^c^	0.004 ^c^	0.289 ^a^	0.005 ^c^	0.003 ^c^	0.103 ^b^	0.007 ^c^	0.001	0.001
*CFB-26*	0.009 ^c^	0.008 ^e^	0.009 ^c^	0.008 ^d^	0.013 ^a^	0.011 ^b^	0.005 ^f^	0.001	0.001
*JG30-KF-CM45*	0.012 ^f^	0.017 ^e^	0.031 ^b^	0.033 ^a^	0.021 ^d^	0.028 ^c^	0.006 ^g^	0.002	0.001
*Deferribacteres (P)*	0.046 ^b^	0.045 ^b^	0.046 ^b^	0.046 ^b^	0.047 ^b^	0.045 ^b^	0.07 ^a^	0.005	0.051
*Schaedleri*	0.008 ^c^	0.008 ^d^	0.011 ^b^	0.006 ^f^	0.013 ^a^	0.007 ^e^	0.008 ^d^	0.000	0.001
*Fusobacteria (P)*	0.484 ^c^	0.007 ^d^	0.001 ^d^	1.309 ^b^	0.004 ^d^	1.926 ^a^	0.004 ^d^	0.020	0.001
*Fusobacterium*	0.051 ^c^	0.003 ^f^	0.002 ^f^	0.138 ^b^	0.017 ^d^	0.204 ^a^	0.007 ^e^	0.000	0.001
*Tenericutes (P)*	0.498 ^a^	0.002 ^g^	0.263 ^b^	0.043 ^e^	0.149 ^d^	0.025 ^f^	0.190 ^c^	0.001	0.001
*RF39*	0.055 ^a^	0.002 ^g^	0.026 ^b^	0.005 ^e^	0.016 ^d^	0.002 ^f^	0.021 ^c^	0.000	0.001
*Verrucomicrobia (P)*	0.001 ^b^	0.001 ^b^	0.004 ^b^	0.005 ^b^	0.005 ^b^	0.004 ^b^	0.159 ^a^	0.001	0.001
*muciniphila*	0.006 ^b^	0.007 ^b^	0.002 ^d^	0.004 ^c^	0.002 ^d^	0.003 ^c^	0.016 ^a^	0.000	0.001
*Thermi (P)*	0.060 ^b^	0.034 ^c^	0.029 ^d^	0.027 ^de^	0.024 ^e^	0.025 ^e^	0.127 ^a^	0.001	0.050
*Thermus*	0.006 ^b^	0.003 ^c^	0.003 ^c^	0.002 ^d^	0.002 ^d^	0.002 ^d^	0.011 ^a^	0.000	0.001

^1^ All data are expressed as the mean ± SEM of the percentage of domain bacteria at taxonomic levels (phylum and family); n = 12 for control group, n = 4 for antibiotic-exposed chicks groups. ^a,b,c,d^ Values among groups are significantly different (*p* < 0.05). ENR-Ad: addition of enrofloxacin; ENR-Nd: no enrofloxacin; DEC-Ad: addition of diclazuril; DEC-Nd: no diclazuril; MIX-Ad: addition of drug mixture; MIX-Nd: no drug mixture; CON: control group.

**Table 2:** Observed OTUs and alpha diversity measures of bacterial communities among control and antibiotic-exposed chick groups.

Alpha Diversity Index	Enrofloxacin (ENR)		Declazuril (DEC)		Mix 1:1 (MIX)		Control	SEM	*p*
	ENR-Ad	ENR-Nd	DEC-Ad	DEC-Nd	MIX-Ad	MIX-Nd	CON		
Observed OTUs	6285.00 ^a^	4527.25 ^b^	6302.75 ^a^	6030.75 ^a^	4413.50 ^b^	5451.50 ^ab^	4115.41 ^b^	275.54	0.001
Simpson	0.94 ^a^	0.91 ^a^	0.96 ^a^	0.94 ^a^	0.80 ^b^	0.94 ^a^	0.94 ^a^	0.020	0.039
Chao1	1516.94 ^a^	1055.91 ^cd^	1511.53 ^a^	1447.44 ^ab^	1140.58 ^bcd^	1319.29 ^abc^	925.53 ^d^	69.70	0.001
ACE	1579.58 ^a^	1071.84 ^bc^	1554.66 ^a^	1483.48 ^a^	1166.55 ^bc^	1331.12 ^ab^	935.19 ^d^	70.18	0.001
Shannon	7.24 ^a^	6.41 ^ab^	7.64 ^a^	7.05 ^a^	5.43 ^a^	6.87 ^b^	6.65 ^ab^	0.270	0.048

^1^ All data are expressed as the mean ± SEM of alpha diversity measures; n = 12 for control group, n = 4 for antibiotic-exposed chicks groups. ^a,b,c,d^ Values among groups are significantly different (*p* < 0.05). ENR-Ad: addition of enrofloxacin; ENR-Nd: no enrofloxacin; DEC-Ad: addition of diclazuril; DEC-Nd: no diclazuril; MIX-Ad: addition of drug mixture; MIX-Nd: no drug mixture; CON: control group.

**Table 3:** Phylogenetic investigation of communities by reconstruction of unobserved states (PICRUSt)-predicted analysis of microbial functions based on KEGG pathway groups, control, and antibiotic-exposed chick groups.

Categories and Levels of KEGG Pathways ^1^	The Relative Abundance for Predicting of Functional Microbiome (%)							SEM	*p*
	ENR-Ad	ENR-Nd	DEC-Ad	DEC-Nd	MIX-Ad	MIX-Nd	CON		
*Cellular Processes*									
Cell growth and death	0.51 ^b^	0.46 ^b^	0.51 ^b^	0.48 ^b^	0.64 ^a^	0.46 ^b^	0.45 ^b^	0.032	0.003
Cell motility	2.63 ^bc^	3.69 ^a^	2.38 ^bc^	2.72 ^bc^	3.18 ^ab^	3.01 ^b^	1.97 ^c^	0.037	0.002
Transport and catabolism	0.19 ^b^	0.21 ^ab^	0.25 ^ab^	0.23 ^ab^	0.28 ^ab^	0.19 ^b^	0.29 ^a^	0.028	0.023
*Environmental Information Processing*									
Membrane transport	13.49 ^a^	12.97 ^a^	12.74 ^ab^	12.6 ^ab^	11.06 ^a^	12.79 ^ab^	13.73 ^a^	0.082	0.074
Signaling molecules and interaction	0.19	0.17	0.18	0.19	0.19	0.19	0.18	0.016	0.973
Signal transduction	1.69	2.06	1.82	1.89	2.02	1.94	1.64	0.146	0.132
*Genetic Information Processing*									
Folding, sorting, and degradation	2.33 ^ab^	2.32 ^ab^	2.32 ^ab^	2.36 ^a^	2.21 ^a^	2.25 ^ab^	2.34 ^ab^	0.043	0.041
DNA replication and repair	8.36 ^a^	8.04 ^b^	7.99 ^bc^	8.34 ^ab^	7.89 ^b^	8.35 ^a^	6.92 ^c^	0.348	0.021
Transcription	2.86	2.74	2.68	2.71	2.74	2.91	2.76	0.094	0.601
Translation	5.41 ^ab^	5.16 ^ab^	5.17 ^ab^	5.37 ^ab^	4.73 ^b^	5.33 ^ab^	5.69 ^a^	0.283	0.035
*Immune Information Processing*									
Immune system diseases	0.06 ^a^	0.05 ^a^	0.05 ^a^	0.06 ^a^	0.04 ^b^	0.05 ^ab^	0.06 ^a^	0.004	0.004
Infectious diseases	0.42 ^a^	0.44 ^a^	0.41 ^ab^	0.43 ^a^	0.47 ^a^	0.44 ^a^	0.4 ^b^	0.021	0.049
Metabolic diseases	0.09 ^ab^	0.09 ^b^	0.08 ^b^	0.08 ^b^	0.08 ^b^	0.09 ^ab^	0.11 ^a^	0.006	0.002
Neurodegenerative diseases	0.17 ^b^	0.22 ^b^	0.23 ^b^	0.22 ^b^	0.44 ^a^	0.17 ^b^	0.16 ^b^	0.050	0.004
*Metabolism Processing*									
Amino acid metabolism	9.56 ^ab^	9.91 ^ab^	9.95 ^ab^	9.74 ^ab^	10.49 ^a^	9.30 ^b^	9.35 ^b^	0.364	0.043
Biosynthesis of secondary metabolites	0.81 ^b^	0.78 ^b^	0.77 ^b^	0.73 ^b^	0.96 ^a^	0.76 ^b^	0.79 ^b^	0.040	0.012
Carbohydrate metabolism	10.53 ^ab^	9.73 ^c^	10.38 ^abc^	9.98 ^bc^	10.4 ^abc^	10.17 ^bc^	10.92 ^a^	0.244	0.003
Energy metabolism	5.35 ^ab^	5.3a ^b^	5.36 ^ab^	5.27 ^ab^	5.55 ^a^	5.17 ^b^	5.41 ^ab^	0.094	0.061
Enzyme families	2.13	2.03	1.96	2.01	2.01	2.11	2.15	0.069	0.199
Glycan biosynthesis and metabolism	1.55 ^b^	1.55 ^b^	1.66 ^ab^	1.68 ^ab^	1.41 ^b^	1.56 ^b^	1.92 ^a^	0.110	0.005
Lipid metabolism	3.07 ^b^	3.2 ^ab^	3.5 ^ab^	3.41 ^a b^	3.63 ^a^	3.03 ^b^	3.14 ^ab^	0.168	0.089
Cofactors and vitamins	4.00	4.05	3.93	3.92	4.08	3.98	3.97	0.120	0.953
Other amino acids	1.67 ^ab^	1.67 ^ab^	1.78 ^ab^	1.71 ^ab^	1.93 ^a^	1.61 ^b^	1.62 ^b^	0.086	0.025
Terpenoids and polyketides	1.86 ^ab^	1.85 ^ab^	2.06 ^a^	1.99 ^ab^	2.09 ^a^	1.74 ^b^	1.75 ^b^	0.097	0.028
Nucleotide metabolism	3.95 ^a b^	3.71 ^b^	3.67 ^b^	3.88 ^a b^	3.42 ^b^	3.87 ^a b^	4.26 ^a^	0.171	0.005
Xenobiotic biodegradation	3.62 ^a^	3.63 ^a^	3.28 ^b^	3.01 ^c^	3.03 ^c^	2.42 ^d^	2.49 ^cd^	0.302	0.047
*Organismal Systems*									
Circulatory system	0.01 ^b^	0.02 ^ab^	0.02 ^b^	0.02 ^b^	0.04 ^a^	0.01 ^b^	0.01 ^b^	0.007	0.018
Digestive system	0.02	0.03	0.03	0.03	0.03	0.03	0.02	0.005	0.139
Endocrine system	0.25 ^b^	0.25 ^b^	0.30 ^b^	0.25 ^b^	0.4 ^a^	0.23 ^b^	0.26 ^b^	0.032	0.015
Excretory system	0.04 ^ab^	0.03 ^ab^	0.03 ^ab^	0.04 ^a^	0.03 ^b^	0.03 ^ab^	0.03 ^ab^	0.004	0.032
Immune system	0.06	0.06	0.05	0.05	0.04	0.06	0.05	0.007	0.609
Nervous system	0.09 ^b^	0.09 ^ab^	0.08 ^b^	0.09 ^b^	0.08 ^b^	0.09 ^b^	0.11 ^b^	0.005	0.001

^1^ All data are expressed as the mean ± SEM of the relative abundance for prediction of the functional microbiomes; ^a,b,c,d^ values among groups are significantly different (*p* <0.05). ENR-Ad: addition of enrofloxacin; ENR-Nd: no enrofloxacin; DEC-Ad: addition of diclazuril; DEC-Nd: no diclazuril; MIX-Ad: addition of drug mixture; MIX-Nd: no drug mixture; CON: control group.

## Supplementary Materials

The following are available online at https://www.mdpi.com/2076-2615/10/5/896/s1, Figure S1: Flowchart showing the selection process for the data included in the analysis. Groups (n = 7); chicks/groups (n = 30); samples/control group (n = 12); samples/treated groups (n = 4) group, Figure S2: (a) Specaccum species accumulation curve showing that the results reflected the rate of increase in new species observed during the continuous sampling of the sample during the overall sampling of the sample. (b) An abundance grade curve visually reflecting the number of high abundances and rare OTUs in the community; the abundance value was converted into the ordinate by Log2 transformation, Figure S3: The total effective sequence amount that passed the quality screening and the indexes were perfectly matched, Figure S4: UPGMA clustering analysis was performed on unweighted and weighted UniFrac distance matrices using QIIME software and visualized using R software among control and antibiotic-exposed chick groups, Figure S5: LEfSe-identified taxa for the control group and six groups of antibiotic-exposed chickens. The classification tree shows the hierarchical relationship of all classification units from the domain to the genus (from the inner circle to the outer circle) in the sample community. The node size corresponds to the average relative abundance of the classification unit, and the letters identify the taxon name that has a significant difference between the groups. (b) LDA scores of taxa enriched in each group are shown as significant at *p* < 0.05, and taxa enriched among seven groups are shown as different colors, Table S1: Statistical number of OTUs at each classification level among the control and antibiotic-exposed chick groups, Table S2: Statistical microbial community annotated at each classification level among control and antibiotic-exposed chick groups, Table S3: Statistics of Metastats comparison test between each pair of control and antibiotic-exposed chicks.

## References

[1] Philippe P., Alzieu J.P., Taylor M.A., Dorchies P. (2014). Comparative efficacy of diclazuril (Vecoxan ^®^) and toltrazuril (Baycox bovis ^®^) against natural infections of Eimeria bovis and Eimeria zuernii in French calves. Vet. Parasitol..

[2] Li J., Hao H., Cheng G., Wang X., Ahmed S., Shabbir M.A.B., Liu Z., Dai M., Yuan Z. (2017). The effects of different enrofloxacin dosages on clinical efficacy and resistance development in chickens experimentally infected with Salmonella Typhimurium. Sci. Rep..

[3] Huang G., Zhang S., Zhou C., Tang X., Li C., Wang C., Tang X., Suo J., Jia Y., Saeed E.A. (2018). Influence of Eimeria falciformis Infection on Gut Microbiota and Metabolic Pathways in Mice. Infect. Immun..

[4] Knaus U.G., Hertzberger R., Pircalabioru G.G., Yousefi S.P., Branco D.S.F. (2017). Pathogen control at the intestinal mucosa - H2O2 to the rescue. Gut Microbes.

[5] Kamada N., Chen G.Y., Inohara N., Núñez G. (2013). Control of pathogens and pathobionts by the gut microbiota. Nat. Immunol..

[6] Harp J.A., Chen W., Harmsen A.G. (1992). Resistance of severe combined immunodeficient mice to infection with Cryptosporidium parvum: The importance of intestinal microflora. Infect. Immun..

[7] Brestoff J.R., Artis D. (2013). Commensal bacteria at the interface of host metabolism and the immune system. Nat. Immunol..

[8] Modi S.R., Collins J.J., Relman D.A. (2014). Antibiotics and the gut microbiota. J. Clin. Investig..

[9] Jakobsson H.E., Cecilia J., Andersson A.F., Maria S.L.K., Jansson J.K., Lars E. (2010). Short-term antibiotic treatment has differing long-term impacts on the human throat and gut microbiome. PLoS ONE.

[10] Tingtao C., Jing Y., Xu F., Hong W., Wei H. (2011). Effects of enrofloxacin on the human intestinal microbiota in vitro. Int. J. Antimicrob. Agents.

[11] Nelson J., Chiller T., Powers J.H., Angulo F. (2007). Fluoroquinolone-resistant Campylobacter species and the withdrawal of fluoroquinolones from use in poultry: A public health success story. Clin. Infect. Dis..

[12] El-Banna H.A., El-Bahy M.M., El-Zorba H.Y., El-Hady M. (2010). Anticoccidial efficacy of drinking water soluble diclazuril on experimental and field coccidiosis in broiler chickens. J. Vet. Med..

[13] Schokker D., Jansman A.J.M., Veninga G., Bruin N.D., Vastenhouw S.A., Bree F.M.D., Bossers A., Rebel J.M.J., Smits M.A. (2017). Perturbation of microbiota in one-day old broiler chickens with antibiotic for 24 hours negatively affects intestinal immune development. BMC Genom..

[14] Carmody R., Gerber G., Jr J.L., Gatti D., Somes L., Svenson K., Turnbaugh P. (2015). Diet Dominates Host Genotype in Shaping the Murine Gut Microbiota. Cell Host Microbe.

[15] Stanisavljević S., Čepić A., Bojić S., Veljović K., Mihajlović S., Đedović N., Jevtić B., Momčilović M., Lazarević M., Mostarica Stojković M. (2019). Oral neonatal antibiotic treatment perturbs gut microbiota and aggravates central nervous system autoimmunity in Dark Agouti rats. Sci. Rep..

[16] Cheng R., Guo J., Pu F., Wan C., Shi L., Li H., Yang Y., Huang C., Li M., He F. (2019). Loading ceftriaxone, vancomycin, and Bifidobacteria bifidum TMC3115 to neonatal mice could differently and consequently affect intestinal microbiota and immunity in adulthood. Sci. Rep..

[17] Blanton L.V., Charbonneau M.R., Salih T., Barratt M.J., Venkatesh S., Ilkaveya O., Subramanian S., Manary M.J., Trehan I., Jorgensen J.M. (2016). Gut bacteria that prevent growth impairments transmitted by microbiota from malnourished children. Science.

[18] Caporaso J.G., Kuczynski J., Stombaugh J., Bittinger K., Bushman F.D., Costello E.K., Fierer N., Peña A.G., Goodrich J.K., Gordon J.I. (2010). QIIME allows analysis of high-throughput community sequencing data. Nat. Methods.

[19] Blaxter M., Mann J., Chapman T., Thomas F., Whitton C., Floyd R., Abebe E. (2005). Defining operational taxonomic units using DNA barcode data. Philos. Trans. R. Soc. Lond. B Biol. Sci..

[20] Chao A., Shen T.-J. (2004). Nonparametric prediction in species sampling. J. Agric. Biol. Environ. Stat..

[21] Simpson E.H. (1949). Measurement of Diversity. Nature.

[22] Shannon C.E. (1948). A Mathematical Theory of Communication. Bell Syst. Tech. J..

[23] Alban R. (2010). Multivariate analyses in microbial ecology. FEMS Microbiol. Ecol..

[24] Lozupone C., Knight R. (2005). UniFrac: A new phylogenetic method for comparing microbial communities. Appl. Environ. Microbiol..

[25] Segata N., Izard J., Waldron L., Gevers D., Miropolsky L., Garrett W.S., Huttenhower C. (2011). Metagenomic biomarker discovery and explanation. Genome Biol..

[26] Langille M.G.I., Zaneveld J., Caporaso J.G., McDonald D., Knights D., Reyes J.A., Clemente J.C., Burkepile D.E., Vega Thurber R.L., Knight R. (2013). Predictive functional profiling of microbial communities using 16S rRNA marker gene sequences. Nat. Biotechnol..

[27] Dethlefsen L., Huse S., Sogin M.L., Relman D.A. (2008). The Pervasive Effects of an Antibiotic on the Human Gut Microbiota, as Revealed by Deep 16S rRNA Sequencing. PLoS Biol..

[28] Strzępa A., Majewskaszczepanik M., Lobo F.M., Wen L., Szczepanik M. (2017). Broad spectrum antibiotic enrofloxacin modulates contact sensitivity through gut microbiota in a murine model. J. Allergy Clin. Immunol..

[29] Bearden D.T., Danziger L.H. (2009). Mechanism of action of and resistance to quinolones. Microb. Biotechnol..

[30] Stanley D., Hughes R.J., Moore R.J. (2014). Microbiota of the chicken gastrointestinal tract: Influence on health, productivity and disease. Appl. Microbiol. Biotechnol..

[31] Swann J.R., Tuohy K.M., Peter L., Brown D.T., Gibson G.R., Wilson I.D., James S., Nicholson J.K., Elaine H. (2011). Variation in antibiotic-induced microbial recolonization impacts on the host metabolic phenotypes of rats. J. Proteome Res..

[32] Liu J.H., Chen Z.L., Yun L.I., Liu Y.W. (2005). Effects of Low Concentration Enrofloxacin on SPF Mice Intestinal Microflora. Sci. Agric. Sin..

[33] Xu Y., Yang H., Zhang L., Su Y., Shi D., Xiao H., Tian Y. (2016). High-throughput sequencing technology to reveal the composition and function of cecal microbiota in Dagu chicken. BMC Microbiol..

[34] Gao P., Ma C., Sun Z., Wang L., Huang S., Su X., Xu J., Zhang H. (2017). Feed-additive probiotics accelerate yet antibiotics delay intestinal microbiota maturation in broiler chicken. Microbiome.

[35] Frost L., Leplae R., Summers A.O., Toussaint A. (2005). Mobile genetic elements: The agents of open source evolution. Nat. Rev. Microbiol..

[36] Modi S.R., Lee H.H., Spina C.S., Collins J.J. (2013). Antibiotic treatment expands the resistance reservoir and ecological network of the phage metagenome. Nature.

[37] D’Costa V.M., King C.E., Kalan L., Morar M., Sung W.W.L., Schwarz C., Froese D., Zazula G., Calmels F., Debruyne R. (2011). Antibiotic resistance is ancient. Nature.

[38] Gautam D., Sommer M.O.A., Oluwasegun R.D., Church G.M. (2008). Bacteria subsisting on antibiotics. Science.

[39] Costello E.K., Keaton S., Les D., Bohannan B.J.M., Relman D.A. (2012). The application of ecological theory toward an understanding of the human microbiome. Science.

