# Supplementary material for: Investigation of the Impacts of Antibiotic Exposure on the Diversity of the Gut Microbiota in Chicks
Source: Animals (Basel). 2020 May 21;10(5):896. doi: 10.3390/ani10050896 (PMC7278382; doi:10.3390/ani10050896)
Supplement: Supplementary file 1 [file animals-10-00896-s001.pdf]

animals

Supplementary data

Investigation of the impacts of antibiotic exposure on the diversity of the gut microbiota in chicks

Abdelmotaleb A. Elokil, Khaled F.M. Abouelezz, Hafiz Ahmad, Yuanhu Pan and Shijun Li

Key Laboratory of Agricultural Animal Genetics, Breeding and Reproduction, Ministry of Education, College of Animal Science and Veterinary Medicine, Huazhong Agricultural University, Wuhan, Hubei 430070, China,

Table S1. Statistical number of OTUs at each classification level among the control and antibiotic-exposed chick groups.

Samples point	Phylum	Class	Order	Family	Genus	Species	Unclassified
ENR1	1501	1501	1500	1295	853	145	0
ENR5	1338	1338	1338	1146	758	66	1
ENR9	1418	1418	1417	1305	858	84	0
ENR13	1291	1291	1290	1185	756	47	0
ENR17	1084	1084	1083	971	542	51	1
ENR21	756	756	756	688	310	56	0
ENR25	1230	1230	1230	1209	792	60	0
ENR29	905	905	905	889	563	53	0
Dec1	1769	1769	1769	1528	927	93	1
Dec5	1051	1051	1049	938	454	84	0
Dec9	1237	1237	1237	1124	753	191	2
Dec13	1496	1496	1495	1360	899	199	2
Dec17	1450	1450	1449	1308	726	142	1
Dec21	1281	1281	1280	1216	918	63	1
Dec25	1635	1635	1634	1552	905	69	0
Dec29	882	882	882	860	572	49	0
Mix1	1331	1331	1331	1133	611	80	1
Mix5	863	863	862	790	557	62	0
Mix9	561	561	561	523	246	27	0
Mix13	1227	1227	1226	1063	552	64	1
Mix17	1239	1239	1239	1103	593	52	0
Mix21	1070	1070	1070	1004	685	49	0
Mix25	1049	1049	1048	1007	643	41	0
Mix29	1449	1449	1449	1377	746	86	0
Control1	878	878	878	747	542	26	0
Control2	811	811	811	665	443	27	0
Control3	906	906	906	764	574	23	0
Control4	1187	1187	1187	952	654	52	0
Control5	969	969	969	816	657	56	0
Control6	872	872	872	768	567	51	0
Control7	878	878	878	756	574	23	0
Control8	1012	1012	1012	860	619	49	0
Control9	926	926	926	798	611	40	0
Control10	823	823	823	755	606	53	0
Control11	767	767	767	663	491	44	0
Control12	869	869	869	860	480	25	0

Table S2. Statistical microbial community annotated at each classification level among control and antibiotic-exposed chick groups.

Samples Point	Phylum	Class	Order	Family	Genus	Species
ENR1	8	18	27	55	83	39
ENR5	9	20	29	55	81	28
ENR9	10	20	30	57	80	31
ENR13	9	17	26	50	62	24
ENR17	10	17	22	42	45	20
ENR21	7	12	18	34	43	13
ENR25	7	15	23	47	52	18
ENR29	7	12	21	39	39	16
Dec1	8	18	28	59	97	39
Dec5	9	19	30	66	94	34
Dec9	6	13	24	45	68	30
Dec13	8	15	23	44	53	25
Dec17	9	19	27	47	70	29
Dec21	8	18	24	45	62	19
Dec25	9	18	27	50	63	22
Dec29	7	14	21	42	41	18
Mix1	8	17	28	63	100	42
Mix5	8	16	22	45	63	25
Mix9	8	16	21	34	43	10
Mix13	9	17	22	40	58	24
Mix17	9	19	28	49	70	24
Mix21	8	15	21	37	48	20
Mix25	6	12	20	39	46	21
Mix29	9	19	31	56	67	26
Control1	9	17	21	39	42	14
Control2	9	16	21	35	33	9
Control3	9	16	21	33	32	8
Control4	9	17	23	35	43	14
Control5	10	19	25	43	50	18
Control6	9	17	22	38	46	13
Control7	9	17	21	42	46	15
Control8	11	20	27	48	65	24
Control9	11	20	29	48	61	29
Control10	11	20	29	51	65	26
Control11	10	19	25	47	55	18
Control12	5	12	19	36	38	13

Table S3. Statistics of Metastats comparison test between each pair of control and antibiotic-exposed chicks.

Groups	Phyla	Genus
Control-DEC.Ad	3	34
Control-DEC.Nd	2	40
Control-ENR.Ad	2	27
Control-ENR.Nd	5	40
Control-Mix.Ad	2	36
Control-Mix.Nd	5	38
DEC.Ad-DEC.Nd	0	4
DEC.Ad-ENR.Ad	0	4
DEC.Ad-ENR.Nd	0	13
DEC.Ad-Mix.Ad	0	5
DEC.Ad-Mix.Nd	1	9
DEC.Nd-ENR.Ad	1	12
DEC.Nd-ENR.Nd	0	3
DEC.Nd-Mix.Ad	0	5

DEC.Nd-Mix.Nd	0	1
ENR.Ad-ENR.Nd	1	11
ENR.Ad-Mix.Ad	0	2
ENR.Ad-Mix.Nd	1	4
ENR.Nd-Mix.Ad	1	6
ENR.Nd-Mix.Nd	0	4
Mix.Ad-Mix.Nd	0	6

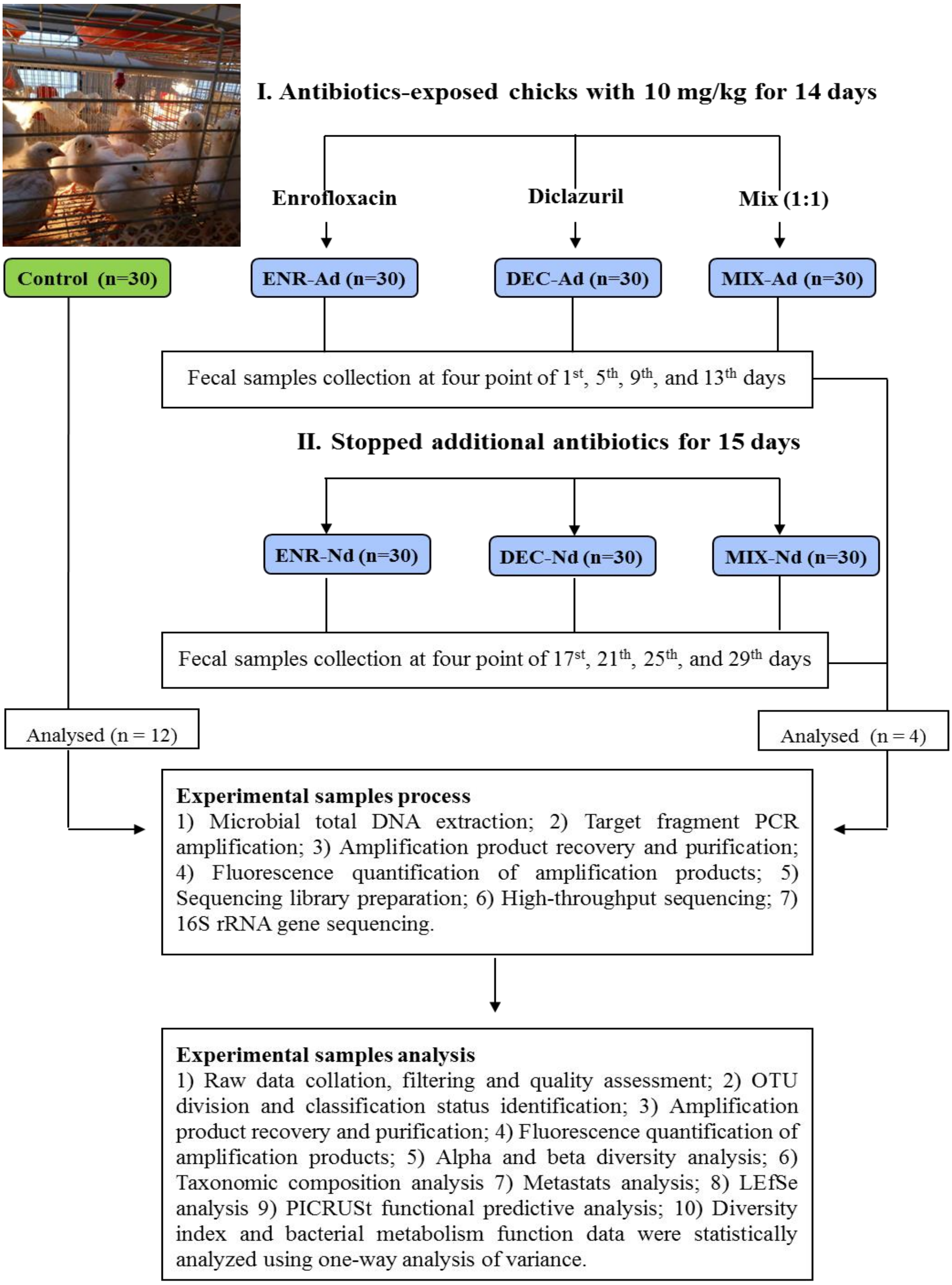

Figure S1. Flowchart showing the selection process for the data included in the analysis. Groups (n = 7); chicks/groups (n = 30); samples/control group (n = 12); samples/treated groups (n = 4) group.

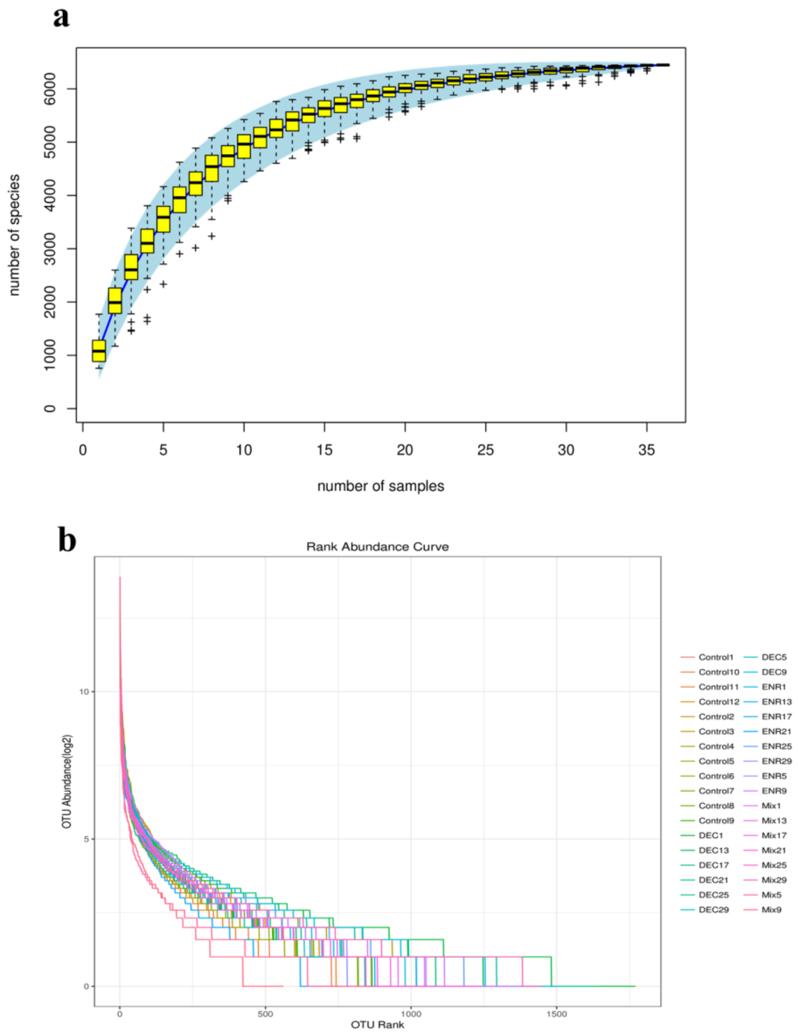

Figure S2. (**a**) Specaccum species accumulation curve showing that the results reflected the rate of increase in new species observed during the continuous sampling of the sample during the overall sampling of the sample. (**b**) An abundance grade curve visually reflecting the number of high abundances and rare OTUs in the community; the abundance value was converted into the ordinate by Log2 transformation.

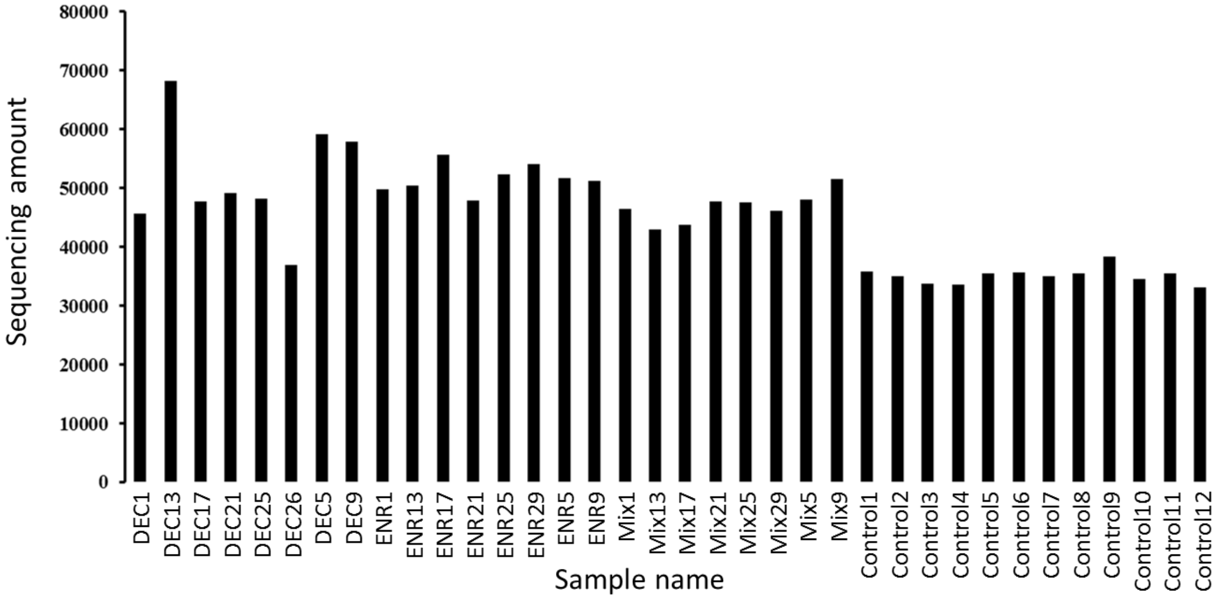

Figure S3. The total effective sequence amount that passed the quality screening and the indexes were perfectly matched.

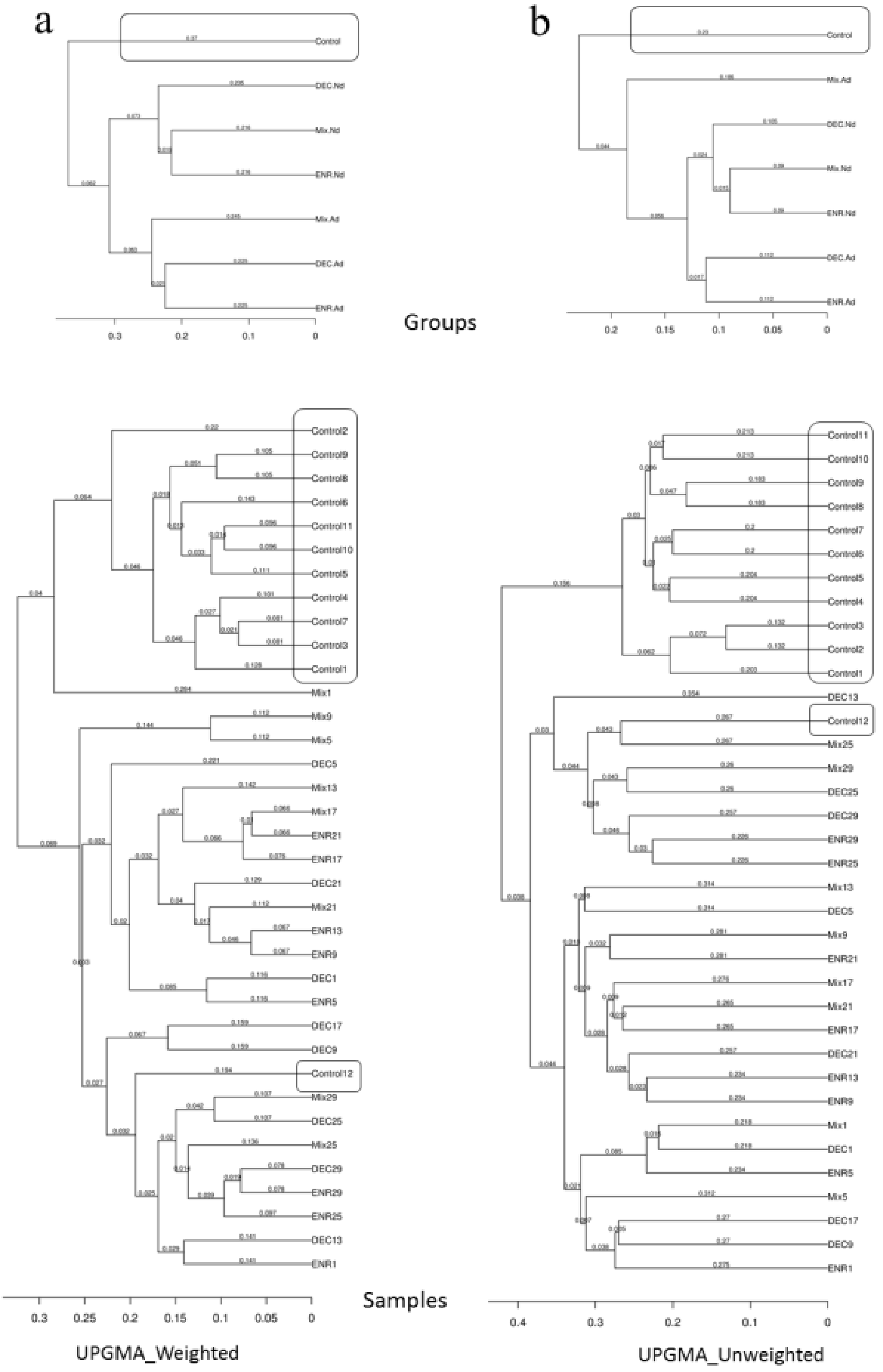

Figure S4. UPGMA clustering analysis was performed on unweighted and weighted UniFrac distance matrices using QIIME software and visualized using R software among control and antibiotic-exposed chick groups.

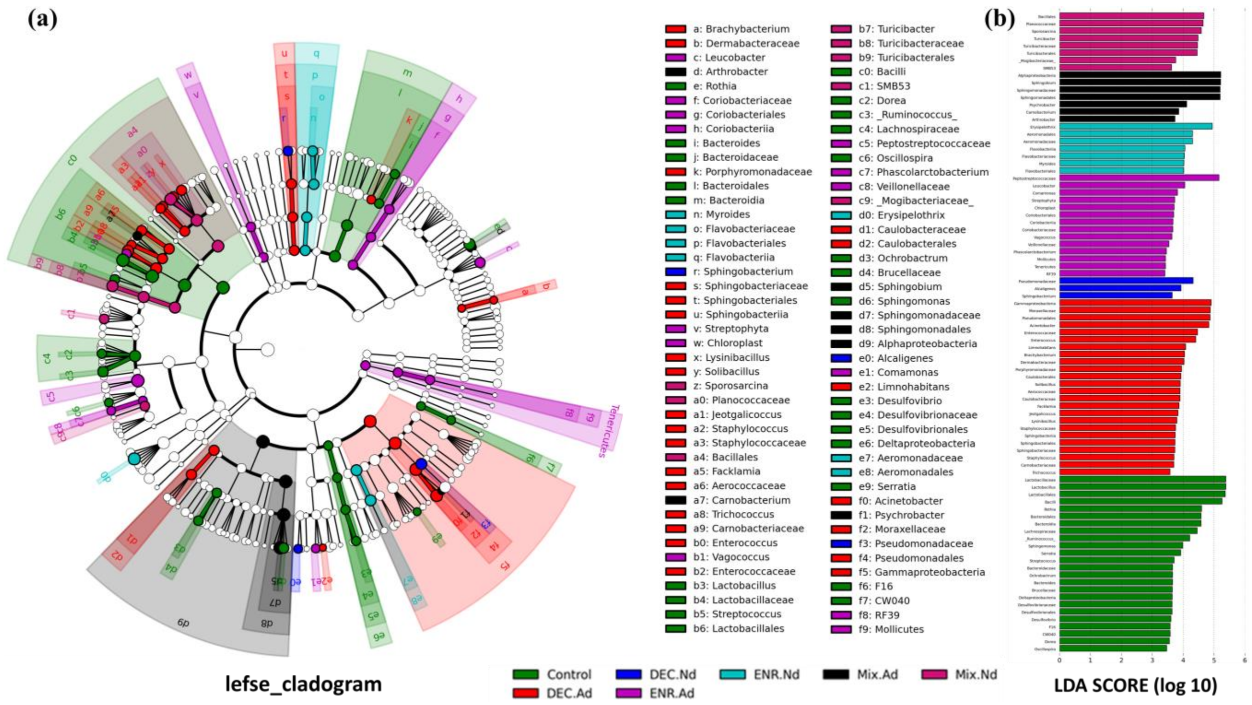

Figure S5. LEfSe-identified taxa for the control group and six groups of antibiotic-exposed chickens. The classification tree shows the hierarchical relationship of all classification units from the domain to the genus (from the inner circle to the outer circle) in the sample community. The node size corresponds to the average relative abundance of the classification unit, and the letters identify the taxon name that has a significant difference between the groups. (b) LDA scores of taxa enriched in each group are shown as significant at $p < 0.05$, and taxa enriched among seven groups are shown as different colors.
